# Race, Class, and the Nursing Home Workforce: Experiences of Staff of Color in High-Minority-Proportion Nursing Homes

**DOI:** 10.1093/geroni/igaa057.2409

**Published:** 2020-12-16

**Authors:** Odichinma Akosionu, Janette Dill, Manka Nkimbeng, Tricia Skarphol, Tetyana Shippee

**Affiliations:** 1 University of Minnesota - School of Public Health, Minneapolis, Minnesota, United States; 2 University of Minnesota, Minneapolis, Minnesota, United States

## Abstract

The long-term services and supports workforce is an important part of delivering quality care for nursing home (NH) residents – and increasingly includes staff who are from diverse communities. Our study captured staff (n=61) perspectives on resident quality of care and quality of life through semi-structured interviews, using thematic analysis in six Minnesota high proportion minority NHs. Findings show that although staff of color are valued for the diversity they contribute to the workforce, and the culturally sensitive care they provide, they are also exposed to discriminatory events. In addition, tensions exist between U.S. and non-U.S. born staff of color in NHs. Overall, staff of color who are lower ranked may feel less empowered. Research is needed to explore the impact of negative and discriminatory exposures on staff wellbeing and related outcomes in addition to the direct and indirect impact on the quality of care delivered to NH residents.

